# Supplementary material for: Prosthesis design of animal models of periprosthetic joint infection following total knee arthroplasty: A systematic review
Source: PLoS One. 2019 Oct 3;14(10):e0223402. doi: 10.1371/journal.pone.0223402 (PMC6776332; doi:10.1371/journal.pone.0223402)
Supplement: S2 File — The search strategies in each database and the inclusion and exclusion of articles. (DOC) [file pone.0223402.s002.doc]

**S1 Search strategy**

**Pubmed**

1. Search (periprosthetic joint infections) AND total knee arthroplasty

2. Search (prosthetic joint infections) AND total knee arthroplasty

3. Search (#1 OR #2)

4. Search (periprosthetic joint infections) AND model

5. Search (prosthetic joint infections) AND model

6. Search (#4 OR #5)

7. Search (periprosthetic joint infections) AND biofilm

8. Search (prosthetic joint infections) AND biofilm

9. Search (#7 OR #8)

10. Search (total knee arthroplasty[Title/Abstract]) AND model[Title/Abstract]

11. Search (#3 OR #6 OR #9 OR #10)

12. Items found [275](https://www.ncbi.nlm.nih.gov/pubmed/?cmd=HistorySearch&querykey=21)9

Embase

1. Search 'prosthetic joint infections' AND 'total knee arthroplasty'
2. Search 'periprosthetic joint infections' AND 'total knee arthroplasty'
3. Search (#1 OR #2)

4. Search 'periprosthetic joint infections' AND 'model'

5. Search 'prosthetic joint infections' AND 'model'

6. Search (#4 OR #5)

7. Search 'periprosthetic joint infections' AND 'biofilm'

8. Search 'prosthetic joint infections' AND 'biofilm'

9. Search (#7 OR #8)

10. Search 'total knee arthroplasty' AND 'model':ti,ab AND ([animal experiment]/lim OR [animal model]/lim)

11. Search (#3 OR #6 OR #9 OR #10)

12. Items found 483

Cochrane Library

1. Search (prosthetic joint infections):ti,ab,kw AND (total knee arthroplasty):ti,ab,kw
2. Search (periprosthetic joint infections):ti,ab,kw AND (total knee arthroplasty):ti,ab,kw
3. Search (#1 OR #2)
4. Search (prosthetic joint infections):ti,ab,kw AND (model):ti,ab,kw
5. Search (periprosthetic joint infections):ti,ab,kw AND (model):ti,ab,kw
6. Search (#4 OR #5)
7. Search (prosthetic joint infections):ti,ab,kw AND (biofilm):ti,ab,kw
8. Search (periprosthetic joint infections):ti,ab,kw AND (biofilm):ti,ab,kw

9. Search (#7 OR #8)

10. Search (total knee arthroplasty):ti,ab,kw AND (model):ti,ab,kw

11. Search (#3 OR #6 OR #9 OR #10)

12. Items found 242

Web of Science (＜1950 - 2019 publication year)

1. Search TS=("prosthetic joint infections" AND "total knee arthroplasty")
2. Search TS=("periprosthetic joint infections" AND "total knee arthroplasty")
3. Search (#1 OR #2)
4. Search TS=("prosthetic joint infections" AND "model")
5. Search TS=("periprosthetic joint infections" AND "model")
6. Search (#4 OR #5)
7. Search TS=("prosthetic joint infections" AND "biofilm")
8. Search TS=("periprosthetic joint infections" AND "biofilm")
9. Search (#7 OR #8)
10. Search TI=("total knee arthroplasty" AND "model")
11. Search (#3 OR #6 OR #9 OR #10)

12. Items found 595.

Wanfang Data

1.检索 主题词扩展&中英文扩展：主题:(“假体周围感染”AND“全膝关节置换”)

2.检索 主题词扩展&中英文扩展：主题:(“假体周围感染”AND“模型”)

3.检索 主题词扩展&中英文扩展：主题:(“假体周围感染”AND“生物膜”)

4.检索 主题词扩展&中英文扩展：主题:(“全膝关节置换”AND“模型”)

5.检索 （#1 OR #2 OR #3 OR #4）*Date:-2019

6.Items found 150.

China National Knowledge Infrastructure

1.检索 SU=假体周围感染*全膝关节置换 OR SU=假体周围感染*模型 OR SU=假体周围感染*生物膜 OR SU=全膝关节置换*模型

2.Items found 70.
